# Supplementary material for: Molecular Dynamics Insight into the CO2 Flooding Mechanism in Wedge-Shaped Pores
Source: Molecules. 2022 Dec 26;28(1):188. doi: 10.3390/molecules28010188 (PMC9821883; doi:10.3390/molecules28010188)
Supplement: Supplementary file 1 [file molecules-28-00188-s001.zip › molecules-2090814-supplementary.pdf]

Supporting Information

Molecular Dynamics Insight into the CO_2 Flooding Mechanism in Wedge-Shaped Pores

Lu Wang [1], Weifeng Lyu [2], Zemin Ji [2], Lu Wang [2], Sen Liu [1], Hongxu Fang [3], Xiaokun Yue [3], Shuxian Wei [1,*], Siyuan Liu [3], Zhaojie Wang [3] and Xiaoqing Lu [3,*]

[1] College of Science, China University of Petroleum, Qingdao 266580, China
[2] State Key Laboratory of Enhanced Oil Recovery, Research Institute of Petroleum Exploration & Development, CNPC, Beijing 100083, China
[3] School of Materials Science and Engineering, China University of Petroleum, Qingdao 266580, China
* Correspondence: wshx@upc.edu.cn (S.W.); luxq@upc.edu.cn (X.L.)

1. Mean Square Displacement (MSD)

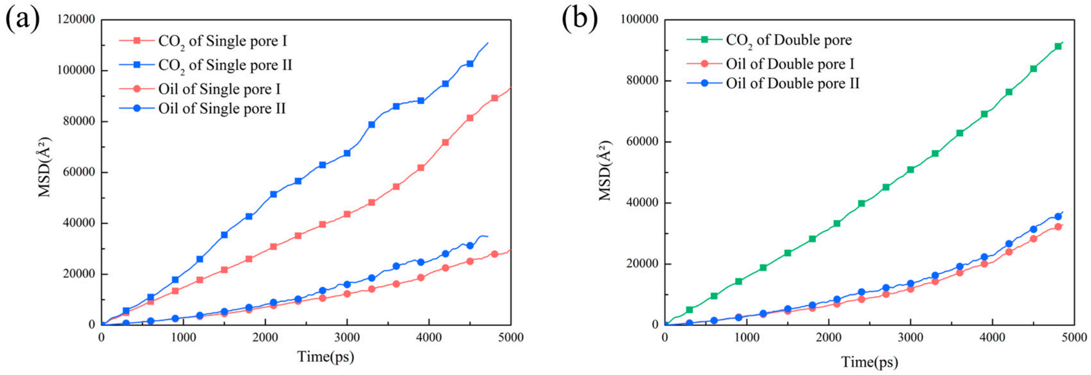

Figure S1. Mean square displacement of CO_2 and oil phase in (**a**) double pores and (**b**) single pores.

2. Hydroxyl coverage of silica surface

We chose single pore II and adjusted its surface hydroxyl coverage to 50% (Figure S2(a)) to examine hydroxyl density's effect on CO_2 flooding in wedge-shaped pores. The density distribution of the oil at various hydroxyl coverage is shown in Figure S2(b), and there is little difference between them. Wedge-shaped pores are influenced by pore shape, and the density figure cannot characterize the adsorption layer as effectively as slit-shaped pores. Therefore, we calculated the interaction energy between oil and rock surface. It can be seen from Figure S2(c), $E_{oil-rock}$ at 50% hydroxylated declined to 0 more quickly than at completely hydroxylated, which suggests that CO_2 can strip oil from the rock surface more quickly at 50% hydroxylated. Thus 50% hydroxylated will result in fewer oil molecules adsorbed on the rock surface, speeding up the rate of CO_2 stripping of oil and improving the efficiency of displacement (Figure S2(d)). However, since all SiO_2 in our systems are constructed in the same way, the degree of hydroxyl coverage does not affect the conclusions of this paper.

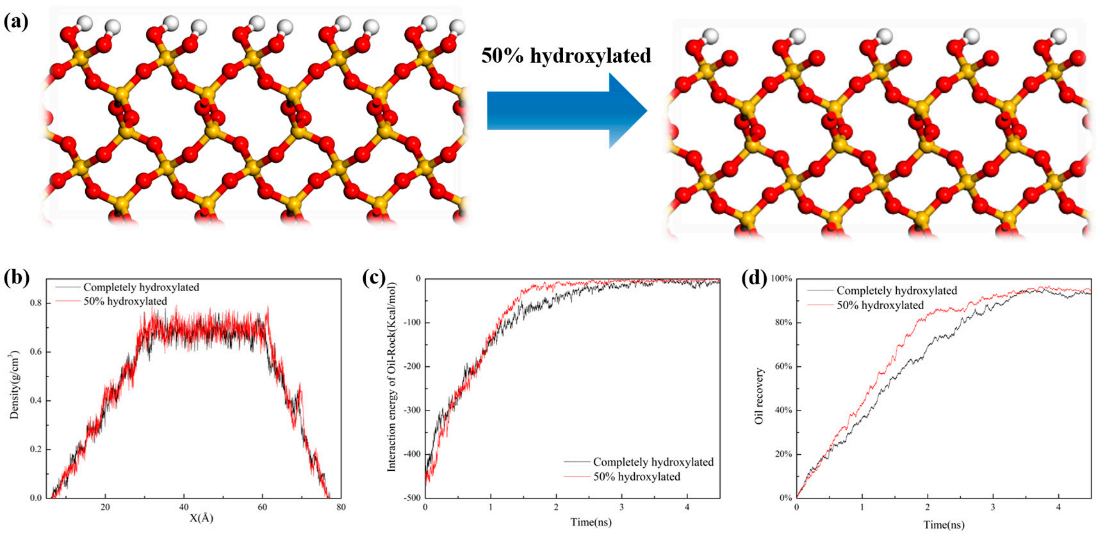

Figure S2. (**a**) 50% hydroxylated silicone surface; (**b**) Density profiles of oil, (**c**) Interaction energy between oil and rock surface, and (**d**) Oil recovery in completely hydroxylated and 50% hydroxylated silicone surface.

3. Different types of alkanes represent the oil phase

To ensure that the results of this study were accurate, we also chose dodecane to represent the oil phase in double pore. The oil transport rate in double pore II is still faster than that in double pore I when the oil is dodecane, and the oil recovery effect is better, as shown by Figure.S3(a) and (b). This is consistent with when octane represents the oil phase. Figure.S3(c) and (d) show that even when the alkane is dodecane, CO_2 and oil molecules still migrate from double pore I to double pore II. "Oil/CO_2 inter-pore migration" and "siphoning" phenomena are still observed to occur in wedge-shaped double pore. As a result, the conclusions of this article are unaffected by the change in the oil phase.

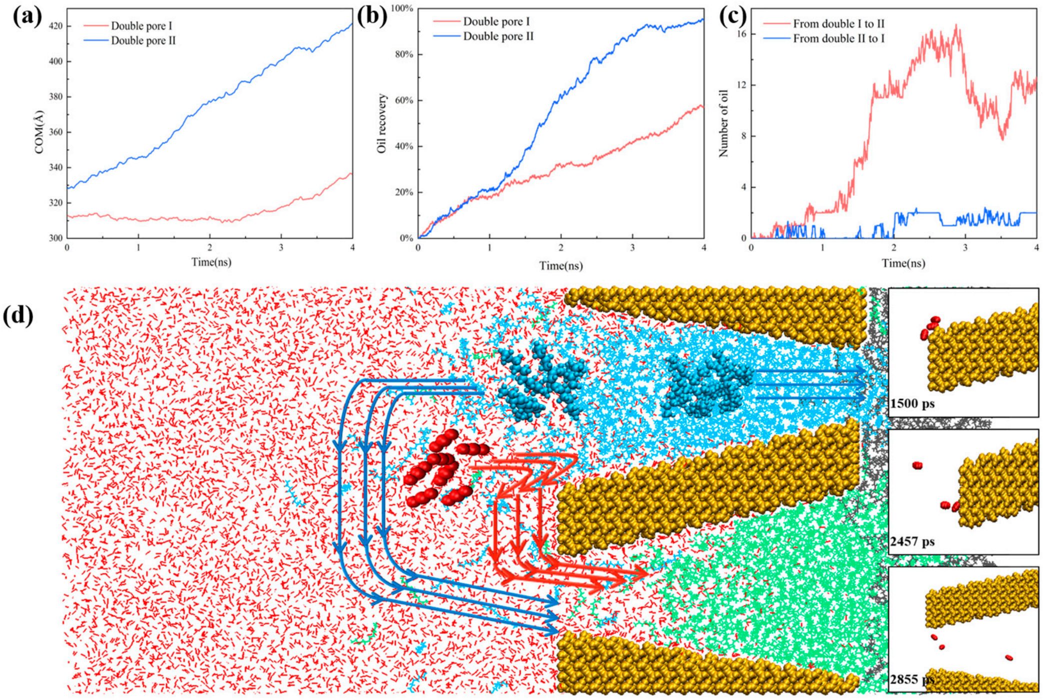

Figure S3. When dodecane represents the oil molecules, (**a**) the COM of oil with time; (**b**) oil recovery in double pore; (**c**) the number of oil molecules migrating from double pore Ⅰ to double pore Ⅱ and from double pore Ⅱ to double pore Ⅰ; (**d**) Left side: streamlines of oil molecules/CO_2 fluid migration from double pore Ⅰ to double pore Ⅱ. Right side: the snapshot of CO_2 migration from double pore Ⅰ to double pore Ⅱ at different times (red: CO_2 molecule; gray, cyan, green: oil phase; orange: shale rock surface).

4. Modeling of slit-shaped pores

For comparison with wedge-shaped pores, 3 nm and 8 nm slit-shaped pores were constructed in Figures S4(a) and S4(b). The length of the rock surface in the Z-direction is 103.5 Å, the thickness in the Y-direction is 28.0 Å, and the width in the X-direction is 23.4 Å. There are 326 randomly placed octane molecules in 3 nm pore and 821 in 8 nm pore.

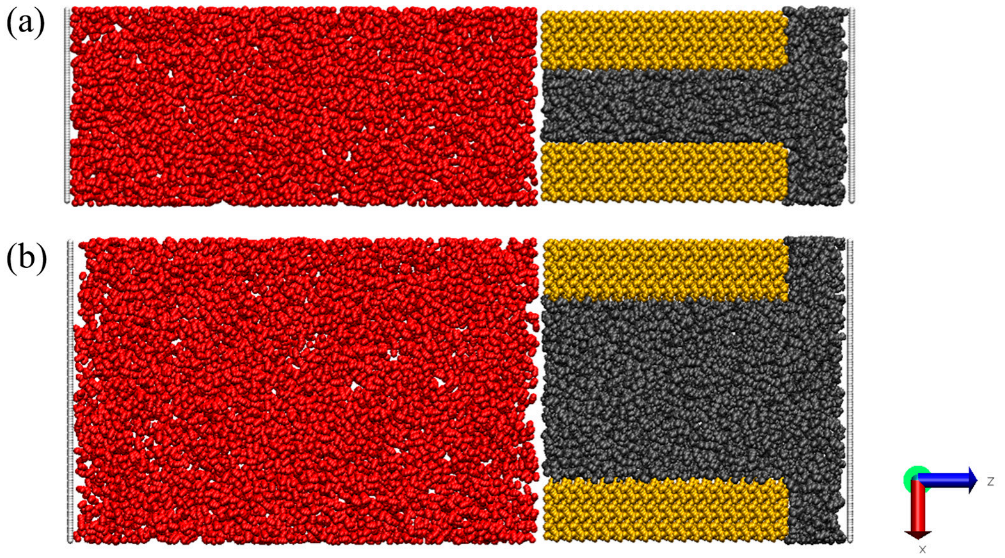

Figure S4. (**a**) 3 nm and (**b**) 8 nm slit-shaped pores (red: CO_2 molecule, gray: oil phase, orange: shale rock surface).

5. Density Comparison

The density of octane in NIST is 0.68982 g/cm3. The average density of the bulk phase octane in the double pore is calculated as 0.68466 g/cm3, 0.68352 g/cm3 for single pore Ⅰ, 0.68020 g/cm3 for single pore Ⅱ, 0.68553 g/cm3 for 3 nm slit-shaped pore, and 0.68477 g/cm3 for 8 nm slit-shaped pore. The calculated results show that the force field is suitable because they diverge just a little from the experimental NIST data.

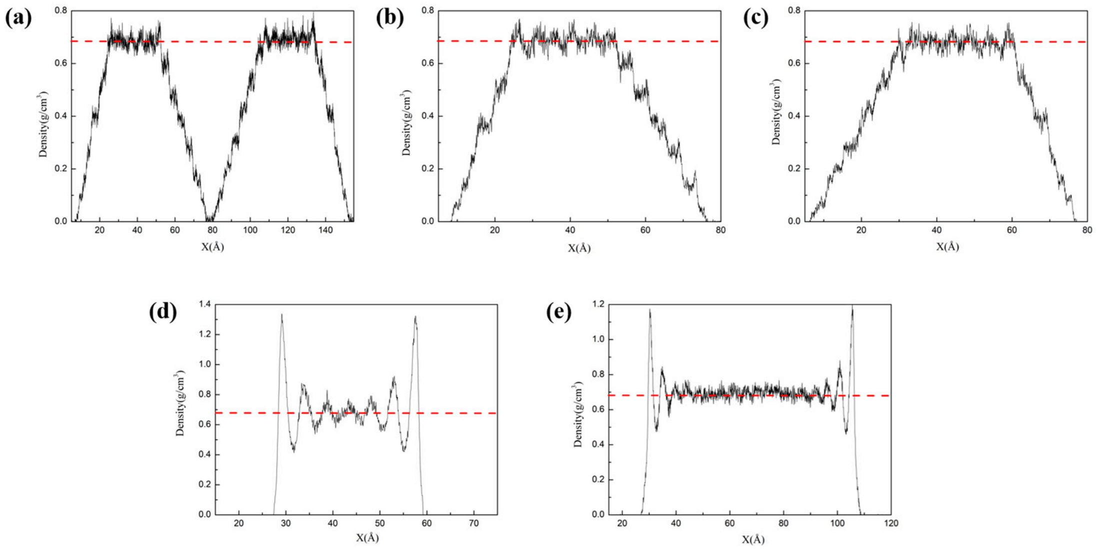

Figure S5. Density profiles of oil in (**a**) double pore; (**b**) single pore Ⅰ; (**c**) single pore Ⅱ; (**d**) 3 nm slit-shaped pore; (**e**) 8 nm slit-shaped pore (the red dashed line is the density of octane in NIST).
